# Comparison of the Performances of Five Primer Sets for the Detection and Quantification of *Plasmodium* in Anopheline Vectors by *Real-Time* PCR

**DOI:** 10.1371/journal.pone.0159160

**Published:** 2016-07-21

**Authors:** V. Chaumeau, C. Andolina, B. Fustec, N. Tuikue Ndam, C. Brengues, S. Herder, D. Cerqueira, T. Chareonviriyaphap, F. Nosten, V. Corbel

**Affiliations:** 1 Centre hospitalier universitaire de Montpellier, Montpellier, France; 2 Maladies Infectieuses et Vecteurs, Ecologie, Génétique, Evolution et Contrôle, Institut de Recherche pour le Développement, Montpellier, France; 3 Centre for Advanced Studies for Agriculture and Food, Institute of Advanced Studies, Faculty of Agriculture, Kasetsart University, Bangkok, Thailand; 4 Shoklo Malaria Research Unit, Mahidol-Oxford Tropical Medicine Research Unit, Faculty of Tropical Medicine, Mahidol University, Mae Sot, Thailand; 5 Institut de Recherche pour le développement, UMR216, Mère et enfant face aux infections tropicales, Paris, France; 6 Communauté d’Universités et d’Etablissements Sorbonne Paris Cité, Faculté de Pharmacie, Paris, France; 7 UMR Intertryp, Institut de Recherche pour le Développement, Montpellier, France; 8 Department of Parasitology, Faculty of Veterinary Medicine, Kasetsart University, Bangkok, Thailand; 9 Department of Entomology, Faculty of Agriculture, Kasetsart University, Bangkok, Thailand; 10 Centre for Tropical Medicine and Global Health, Nuffield Department of Medicine, University of Oxford, Oxford, United Kingdom; Kansas State University, UNITED STATES

## Abstract

Quantitative real-time polymerase chain reaction (qrtPCR) has made a significant improvement for the detection of *Plasmodium* in anopheline vectors. A wide variety of primers has been used in different assays, mostly adapted from molecular diagnosis of malaria in human. However, such an adaptation can impact the sensitivity of the PCR. Therefore we compared the sensitivity of five primer sets with different molecular targets on blood stages, sporozoites and oocysts standards of *Plasmodium falciparum* (Pf) and *P*. *vivax* (Pv). Dilution series of standard DNA were used to discriminate between methods at low concentrations of parasite and to generate standard curves suitable for the absolute quantification of *Plasmodium* sporozoites. Our results showed that the best primers to detect blood stages were not necessarily the best ones to detect sporozoites. Absolute detection threshold of our qrtPCR assay varied between 3.6 and 360 Pv sporozoites and between 6 and 600 Pf sporozoites per mosquito according to the primer set used in the reaction mix. In this paper, we discuss the general performance of each primer set and highlight the need to use efficient detection methods for transmission studies.

## Introduction

Malaria is a vector-borne disease transmitted to human through the bite of a female *Anopheles* infected with *Plasmodium*. Five species are responsible for malaria in human, namely *Plasmodium falciparum* (Pf), *P*. *vivax* (Pv), *P*. *ovale* (Po), *P*. *malariae* (Pm) and *P*. *knowlesi* (Pk) [1, 2]. Inaccurate detection of *Plasmodium* in the vector (either false negative or false positive reaction) can have considerable consequences on the estimation of the intensity of malaria transmission and then bias the understanding of malaria epidemiology [3–6]. In the context of deployment of global effort towards malaria control and elimination, it is of primary importance to use reliable diagnostic tools for the detection of *Plasmodium* in anopheline vectors.

In routine practice, three methods are used to detect *Plasmodium* in the vectors: microscopic observation of dissected salivary glands [7, 8], enzyme linked immuno-sorbent assays targeting the circumsporozoite protein (ELISA-CSP) [7, 9–15] and polymerase chain reaction (PCR) [7, 10, 15–20]. Compared to other methods, PCR has significantly improved the sensitivity and the specificity of the detection and allowed for an accurate identification of the plasmodial species [3, 6, 21, 22]. The assay sensitivity and specificity depend on a variety of factors interacting together such as the conditions of the PCR (essentially the hybridization temperature for primers annealing, and the MgCl_2_, primers and DNA polymerase concentrations), the DNA target, the primers and the matrix of the reaction [23]. Therefore, quantitative real-time PCR (qrtPCR) is not necessarily more sensitive than conventional PCR (cvPCR), ELISA-CSP or microscopy [23]. However, it is commonly admitted that qrtPCR technology is a progress regarding its ability to quantify the number of PCR DNA target (hence the amount of parasite in a biological sample), reduce the labour load and limit the risk of contamination [23, 24].

Most of the literature available on the molecular detection of *Plasmodium* in mosquitoes has been adapted from assays that were developed for the diagnosis of malaria parasites in humans [19, 20, 25–29]. However the sensitivity and specificity of an assay developed on blood samples may vary when applied to malaria vectors because of non-specific amplifications generating false positive [28, 29] and/or inhibition of the PCR due to inhibitory components that may remain in the DNA template after the extraction step [28–30]. Moreover, differences in the composition of the matrix can affect the efficiency of the PCR and impact on the detection threshold [23, 31].

In this study we compared the performances of a qrtPCR assay optimised for five primer sets selected from the literature [32–36] because *(i)* they proved to be efficient to detect human malaria parasites and *(ii)* they amplify different PCR DNA targets of the nuclear (18S ssuRNA genes) or mitochondrial (COX I, COX III, other non-coding sequence) genome. Dilution series of standard DNA extracts (Pf and Pv sporozoites, oocysts and blood stages) were used to assess detection limits of the different methods and to produce standard curves for the absolute quantification of sporozoites in malaria vectors.

This work aims at providing recommendations on the most efficient molecular technique to use for the detection and quantification of *Plasmodium* in anopheline vectors.

## Material & Methods

### Standard samples of *Plasmodium falciparum* and *Plasmodium vivax*

#### Blood stages

Blood samples were collected from patients attending the Shoklo Malaria Research Unit (SMRU) clinics with a clinical episode of Pf or Pv malaria. DNA was extracted using the DNEasy kits® (Qiagen) according to the manufacturer’s instructions for human blood (100μl of blood was extracted and eluted in 100μl of elution buffer such a way as 1μl of DNA extract corresponded to 1μl of blood).

#### Heads/thoraxes (containing sporozoites) and abdomens (containing oocysts) from experimentally infected *Anopheles*

Batches of *Anopheles cracens* reared at the SMRU were experimentally infected by membrane feeding as previously described [37]. Fifteen days after the infective blood meal, infected specimens were killed by freezing and cut in two parts in order to separate the head/thorax (containing putatively sporozoites) from the abdomen (containing putatively oocysts). The samples were crushed in 200μl of cetyltrimethylammonium bromide (CTAB) solution 2% (TrisHCl pH = 8, 20mM; EDTA 10mM; NaCL, 1.4 mM; N-cetyl-N,N,N,-trimethyl ammonium bromide 2%) with a TissueLyser II® (Qiagen). The samples were warmed at 65°C for 5 minutes and 200μl of chloroform were added. The organic phase was collected and DNA was precipitated with 200μl of isopropanol. After centrifugation at 20,000 g for 15 minutes, the pellet was washed with 70% ethanol and suspended in 40 μl of PCR water.

#### Calibrated suspension of *Plasmodium vivax* and *Plasmodium falciparum* sporozoites

The salivary glands of 100 infected mosquitoes were pooled and crushed in a 1.5 ml tube (Eppendorf) containing 50 μl of RPMI® medium (Sigma) and the concentration of the suspension was estimated by inverted microscopy using a KOVA slide [37]. Three different vials of Pv sporozoites were produced and contained 288,000, 330,750 and 460,600 parasites respectively (mean of 360,000 sporozoites per vial). One vial of Pf sporozoites was produced and contained 60,000 parasites. DNA was extracted with the CTAB protocol as described previously.

### qrtPCR assay and primers

Five primer sets suitable for the detection of *Plasmodium* were selected from the literature [32–35, 38] and adapted to qrtPCR technology using universal-thermocycling protocol and intercalating dye as a detection method. The sequences of the primers are presented in the Table 1 and were produced by BioDesign (Thailand). All experiments were performed on a CFX-96® (Biorad) machine; reactions were conducted in 9μl of EVAGreen qPCR Mix Plus® (Euromedex); 1μl of DNA template was used in a total reaction volume of 10μl; the same thermocycling protocol was applied to all primer sets (95°C for 15 minutes followed by 45 amplification cycles at 95°C for 15 seconds, appropriate annealing temperature for 15 seconds and 72°C for 20 seconds); characterisation of the PCR product was performed using the melt curve analysis of the amplicons (95°C for 15 seconds, 68°C for 1 minute, 80°C for 15 seconds, 60°C for 15 seconds, then 60°C to 90°C with an increment of 0.2°C per second). The primer set II was used in duplex with the primers Pf1, Pf2, Pv1 and Pv2 in the same reaction mix. The primer set V was used in duplex with the primers VIV-F, VIV-R, FAL-F and FAL-R in the same reaction mix (the primers OVA-F and OVA-R were not included in the reaction mix). The optimal hybridization temperature for primers annealing and the concentrations of primers and MgCl_2_ were determined in a single cross-experiment for each primer set using the gradient mode of the machine. Appropriate positive (*Plasmodium* DNA) and negative controls (water) were included in each experiment. As we used the same amplification protocol, combined with an optimization of the reaction conditions, the primer set, the primer concentration and the annealing temperature were the only factor affecting the sensitivity of the PCR.

**Table 1:** Sequences of the primers used in the study and corresponding references.

Primer set	Ref.	Name	Sequence	PCR DNA target	Target species
I	[33]	Forward	5’-TAGCCGACAAGGAATTTTGC-3’	ncMS	Pspp
		Reverse	5’-CCTTGAATGGAGCACTGGAT-3’		
II	[34]	Pf1	5’-CCTGCATTAACATCATTATATGGTACATCT-3’	COX I	Pf
		Pf2	5’-GATTAACATTCTTGATGAAGTAATGATAATACCTT-3’		
		Pv1	5’-AAGTGTTGTATGGGCTCATCATATG-3’	COX III	Pv
		Pv2	5’-CAAAATGGAAATGAGCGATTACAT-3’		
III	[36]	Pspp1	5'-AGTTACGATI'AATAGGAGTAG-3’	18S ssuRNA genes	Pspp
		Pspp2	5'-CCAAAGACTI'TGATTTCTCAT-3'		
IV	[32]	PL1473F18	5’-TAACGAACGAGATCT TAA-3’	18S ssuRNA genes	Pspp *
		PL1679R18	5’-GTTCCTCTAAGAAGCTTT-3’		
V	[35]	FAL-F	5’-CTTTTGAGAGGTTTTGTTACTTTGAGTAA-3’	18S ssuRNA genes	Pf
		FAL-R	5’-TATTCCATGCTGTAGTATTCAAACACAA-3’		
		VIV-F	5’-ACGCTTCTAGCTTAATCCACATAACT-3’	18S ssuRNA genes	Pv
		VIV-R	5’-ATTTACTCAAAGTAACAAGGACTTCCAAGC-3’		

**Ref.**, reference; **ncMS**, non-coding mitochondrial sequence; **COX**, cyclo-oxygenase; **Pf**, *Plasmodium falciparum*; **Pspp**, *Plasmodium spp*.; **Pv**, *Plasmodium vivax*.

* identification of the plasmodial species is possible through the melt-curve analysis of the PCR product.

### Serial dilution assays

DNA extracts from uninfected samples were pooled in order to obtain a homogeneous negative 1X DNA matrix from *Anopheles* abdomen or head/thorax and human blood. Ten-fold dilutions series of standard DNA extracts of *Plasmodium* (according to the preliminary CP) were done in the appropriate matrix. Each vial was aliquoted and stored at -20°C during one month (duration of the study) in order to avoid multiple defrosting.

### Data analysis

#### Scoring system to assess the proportion of positive reactions at low concentrations of parasite

Dilution series from head/thorax (containing sporozoites), abdomen (containing oocysts) and blood (containing blood stages) were tested twice (6 reactions in each experiment) yielding 12 PCR results per dilution. In order to facilitate the comparison of different primers, we used a scoring system as described by Sterkers *et al*. [39]. The score consisted of calculating a ratio of the number of positive reactions to the total number of reactions performed at low parasitic concentrations (*i*.*e*. dilutions at which at least one primer sets yielded < 12 positive reactions). This score, reflecting the “proportion of positive reactions for low concentrations of parasite”, was calculated for each serial dilution experiment and for each primers set. The score values were compared using pairwise Chi-square test adjusted with Bonferroni’s correction for multiple comparisons and Yate’s correction when observed frequencies were less than five.

#### Absolute quantification of sporozoites using calibrated standards

Serial dilutions of DNA extracts from calibrated sporozoites standards were tested in triplicate yielding 9 PCR results per dilution. Crossing-point (CP) values were determined using the regression algorithm of the analysis software of the PCR device (CFX Biorad Manager version 3.0®, Biorad) and used to elaborate the standard curves. The best fit-line and the subsequent values of the slope and y-intercept were obtained using least-square analysis of the linear portion of each curve (Pearson’s coefficient r^2^>0.990). The PCR efficiency (EFF) was calculated from the formula EFF = 10^(-1/slope)^–1. Accuracy and repeatability of the measure were estimated using intra- and inter-assay standard deviation (SD). The limit of detection (LOD) was defined as the highest dilution with ≥50% of positive reactions (*i*.*e*. amplification of the PCR DNA target). Concentrations were expressed in number of *Plasmodium falciparum* or *Plasmodium vivax* genome (Pfg or Pvg) equivalents per reaction tube. Considering our protocol, one genome equivalent per reaction tube corresponds to 40 sporozoites per mosquito prior to DNA extraction.

### Ethics approval

The protocol for blood collection and analysis has been approved by the Oxford Tropical Research Ethics Committee (1015–13, dated 29 Apr 2013). All participants provided their written consent to participate in this study. This consent procedure was approved by the ethics committee.

## Results

### Optimal conditions for the PCR

**Fig 1:**
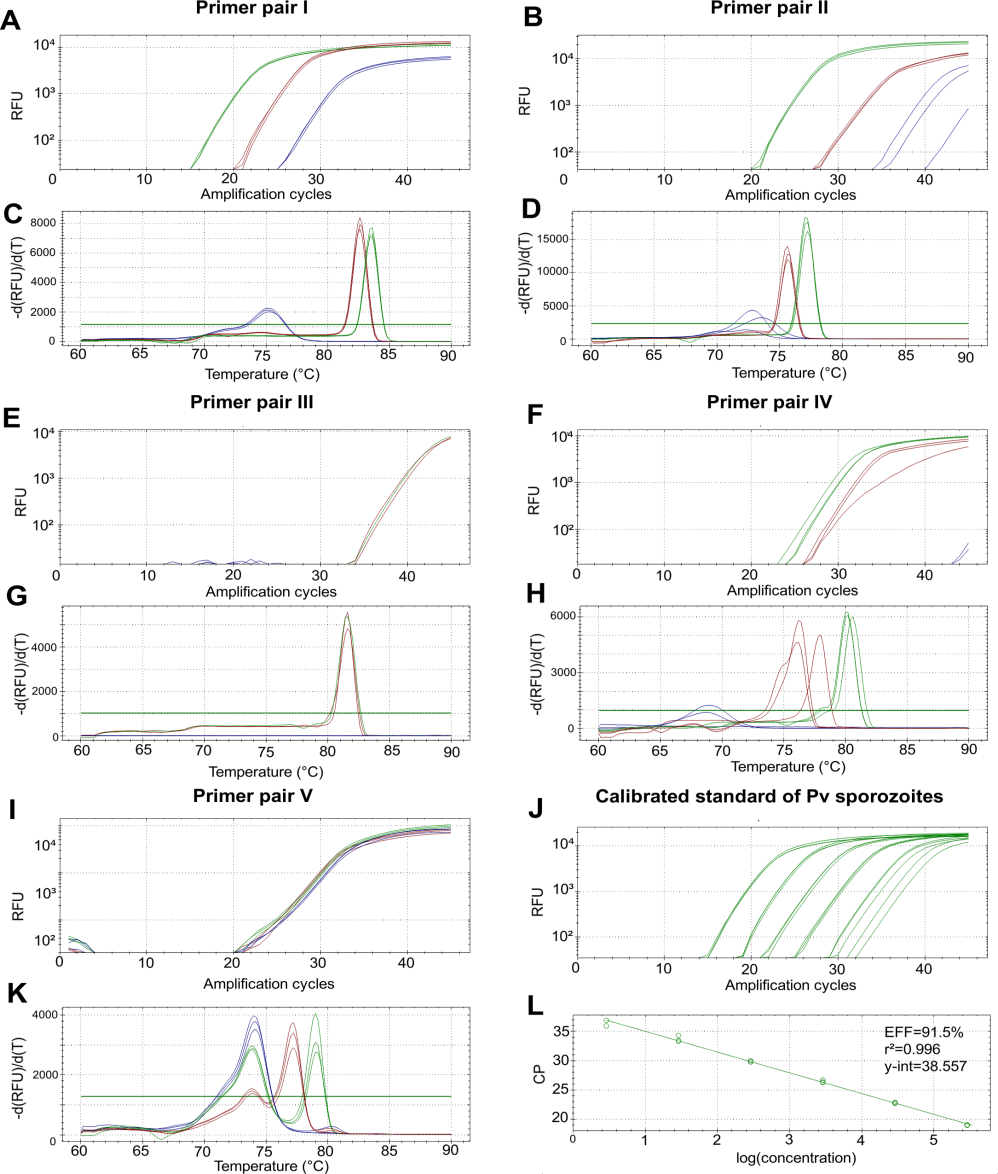
Typical amplification curves, melt curves and standard curves generated with the five primer sets. (A,B,E,F and I) Amplification curves generated by the real-time measurement of the fluorescent signal at the end of each amplification cycle; (C,D,G,H, and K) Melt curves analysis of the PCR product; (J and L) Amplification curves and standard curve generated during the assessment of primer set II on dilution series of calibrated *Plasmodium* vivax sporozoites standard (vial 1), demonstrating the linear relationship between the logarithm of the parasitic concentration and the CP value. Brown and green lines represent *Plasmodium falciparum* and *Plasmodium vivax* standards respectively; blue lines represent the non-template negative control.

Typical amplification and melt curves obtained with Pf and Pv standards are illustrated in the Fig 1, panels A to J. All primer sets accurately amplified positive Pf and Pv samples and three of them were able to identify the species of *Plasmodium* (primer sets II, IV and V).

The reaction conditions (essentially the hybridization temperature for primers annealing and the concentration of MgCl_2_ and primers) have a great influence on the sensitivity and specificity of the PCR assay. It was therefore essential to optimize the reaction conditions and to determine the efficiency of the PCR before comparing the different primers. The optimal conditions of the reaction (adapted to the CFX-96 device) and the corresponding efficiency are presented in the Table 2. The efficiency was >80% and <110% for the primer sets I, II, III and IV hence reflecting a good optimisation of the reaction conditions. A lot of primer-dimers was however detected with the primer set V (Fig 1, panel J), thus it was not possible to determine the efficiency of the reaction.

**Table 2:** Optimal reaction conditions and corresponding efficiencies using the CFX-96® (Biorad) device.

Primer set	Annealing temperature (°C)	MgCl_2_ concentration (mM)	Primers concentration (nM)	%EFF ^a^ (Pf)	%EFF ^a^ (Pv)
I	58	2.5	250 each	ND ^b^	110
II	60	2.5	300 each	84	92
III	58	2.5	500 each	96	110
IV	54	2.5	250 each	97	101
V	60	2.5	400 each	ND ^c^	ND ^c^

**ND,** not determined; **Pf**, *Plasmodium falciparum*; **Pv**, *Plasmodium vivax;* **EFF**, efficiency of the PCR.

^a^ %EFF: efficiency (EFF) of the PCR calculated with the formula EFF = 10^(-1/slope)^ - 1 and expressed as a percentage. An efficiency of 100% corresponds to a slope of -3.32 and means that the number of amplicons doubles after each cycle of amplification. Efficiency was calculated from serial dilutions of calibrated Pf and Pv sporozoite standards.

^b^ Due to the lack of sample, it was not possible to determine the PCR efficiency of the primer pair I with the Pf sporozoite standard.

^c^ Due to the presence of primer-dimers, it was not possible to determine the PCR efficiency with primer set V.

### Comparison of the sensitivity of the five primer sets on sporozoites, oocysts and blood stage standards of *Plasmodium falciparum* and *Plasmodium vivax*

As expected, the number of positive reactions was 100% (12/12) with all primers at high concentrations of parasite whereas it progressively declined as the dilution factor increased (Table 3). At low concentrations of parasite, a significant disparity in the score values was observed, supporting that this approach is efficient to discriminate between different primers. The results of the pairwise Chi-square tests adjusted with Bonferroni’s correction are presented in the S1 Table. On Pf standards, the best score values were obtained with the primer sets I, II and IV for sporozoites, oocysts and blood stages respectively. Regarding the detection of Pv, the best results were obtained with the primer sets I and II which had similar score values on all standards. The score values obtained with the primer set V was significantly lower than the score values obtained with the other primers on all *Plasmodium* standards.

**Table 3:** Results of the assessment of each primer set on sporozoites, oocysts and blood stages standards.

Standard sample	Species	Primer set	Dilution																				Score^b^	
			ND		10^−1^		10^−2^		10^−3^		10^−4^		10^−5^		10^−6^		10^−7^		10^−8^		10^−9^		nb	%
			nb^a^	%a	nb	%	Nb	%	nb	%	nb	%	nb	%	nb	%	nb	%	nb	%	nb	%		
Head/Thorax (sporozoites)	Pf	I	12/12	100	12/12	100	12/12	100	**12/12**	**100**	**12/12**	**100**	**10/12**	**83**	**2/12**	**17**	-	-	-	-	-	-	36/48	75
		II	12/12	100	12/12	100	12/12	100	**12/12**	**100**	**12/12**	**100**	**9/12**	**75**	**3/12**	**25**	-	-	-	-	-	-	36/48	75
		III	12/12	100	12/12	100	11/12	92	**11/12**	**92**	**8/12**	**67**	**3/12**	**25**	**0/12**	**0**	-	-	-	-	-	-	22/48	46
		IV	12/12	100	12/12	100	12/12	100	**12/12**	**100**	**8/12**	**67**	**4/12**	**33**	**1/12**	**8**	-	-	-	-	-	-	25/48	52
		V	12/12	100	12/12	100	12/12	100	**12/12**	**100**	**0/12**	**0**	**0/12**	**0**	**0/12**	**0**	-	-	-	-	-	-	12/48	25
	Pv	I	12/12	100	12/12	100	12/12	100	12/12	100	**12/12**	**100**	**7/12**	**58**	**4/12**	**33**	0/12	0	-	-	-	-	23/36	64
		II	12/12	100	12/12	100	12/12	100	12/12	100	**12/12**	**100**	**5/12**	**42**	**1/12**	**8**	0/12	0	-	-	-	-	18/36	50
		III	12/12	100	12/12	100	12/12	100	12/12	100	**8/12**	**67**	**1/12**	**8**	**1/12**	**8**	0/12	0	-	-	-	-	10/36	28
		IV	12/12	100	12/12	100	12/12	100	12/12	100	**7/12**	**58**	**3/12**	**25**	**1/12**	**8**	0/12	0	-	-	-	-	11/36	31
		V	12/12	100	12/12	100	12/12	100	0/12	0	**0/12**	**0**	**0/12**	**0**	**0/12**	**0**	0/12	0	-	-	-	-	0/36	0
Abdomen (oocysts)	Pf	I	12/12	100	12/12	100	12/12	100	**12/12**	**100**	**7/12**	**58**	**5/12**	**42**	**1/12**	**8**	-	-	-	-	-	-	25/48	52
		II	12/12	100	12/12	100	12/12	100	**12/12**	**100**	**7/12**	**58**	**4/12**	**33**	**1/12**	**8**	-	-	-	-	-	-	24/48	50
		III	12/12	100	12/12	100	12/12	100	**9/12**	**75**	**3/12**	**25**	**1/12**	**8**	**0/12**	**0**	-	-	-	-	-	-	13/48	27
		IV	12/12	100	12/12	100	12/12	100	**8/12**	**67**	**4/12**	**33**	**1/12**	**8**	**0/12**	**0**	-	-	-	-	-	-	13/48	27
		V	12/12	100	12/12	100	10/12	83	**0/12**	**0**	**0/12**	**0**	**0/12**	**0**	**0/12**	**0**	-	-	-	-	-	-	0/48	0
	Pv	I	12/12	100	12/12	100	12/12	100	**12/12**	**100**	**9/12**	**75**	**4/12**	**33**	**1/12**	**8**	**0/12**	**0**	-	-	-	-	26/60	43
		II	12/12	100	12/12	100	12/12	100	**12/12**	**100**	**12/12**	**100**	**6/12**	**50**	**3/12**	**25**	**2/12**	**17**	-	-	-	-	35/60	58
		III	12/12	100	12/12	100	12/12	100	**7/12**	**58**	**4/12**	**33**	**1/12**	**8**	**0/12**	**0**	**0/12**	**0**	-	-	-	-	12/60	20
		IV	12/12	100	12/12	100	12/12	100	**11/12**	**92**	**6/12**	**50**	**0/12**	**0**	**0/12**	**0**	**0/12**	**0**	-	-	-	-	17/60	28
		V	12/12	100	11/12	92	0/12	0	**0/12**	**0**	**0/12**	**0**	**0/12**	**0**	**0/12**	**0**	**0/12**	**0**	-	-	-	-	0/60	0
Blood (blood stages)	Pf	I	12/12	100	12/12	100	12/12	100	12/12	100	**12/12**	**100**	**10/12**	**83**	**3/12**	**25**	**0/12**	**0**	-	-	-	-	25/48	52
		II	12/12	100	12/12	100	12/12	100	12/12	100	**12/12**	**100**	**11/12**	**92**	**2/12**	**17**	**1/12**	**8**	-	-	-	-	26/48	54
		III	12/12	100	12/12	100	12/12	100	12/12	100	**6/12**	**50**	**3/12**	**25**	**0/12**	**0**	**0/12**	**0**	-	-	-	-	9/48	19
		IV	12/12	100	12/12	100	12/12	100	12/12	100	**8/12**	**67**	**2/12**	**17**	**1/12**	**8**	**0/12**	**0**	-	-	-	-	11/48	23
		V	12/12	100	12/12	100	12/12	100	10/12	83	**1/12**	**8**	**0/12**	**0**	**0/12**	**0**	**0/12**	**0**	-	-	-	-	1/48	2
	Pv	I	12/12	100	12/12	100	12/12	100	12/12	100	12/12	100	12/12	100	**8/12**	**67**	**3/12**	**25**	**1/12**	**8**	0/12	0	12/36	33
		II	12/12	100	12/12	100	12/12	100	12/12	100	12/12	100	12/12	100	**11/12**	**92**	**3/12**	**25**	**3/12**	**25**	0/12	0	17/36	47
		III	12/12	100	12/12	100	12/12	100	12/12	100	12/12	100	12/12	100	**11/12**	**92**	**1/12**	**8**	**0/12**	**0**	0/12	0	12/36	33
		IV	12/12	100	12/12	100	12/12	100	12/12	100	12/12	100	12/12	100	**11/12**	**92**	**5/12**	**42**	**3/12**	**25**	0/12	0	19/36	53
		V	12/12	100	12/12	100	12/12	100	12/12	100	12/12	100	0/12	0	**0/12**	**0**	**0/12**	**0**	**0/12**	**0**	0/12	0	0/36	0

**ND**, not diluted; **Pf**, *Plasmodium falciparum*; **Pv**, *Plasmodium vivax*.

^a^ number (nb) or percentage (%) of positive reactions over the total number of reaction performed.

^b^ score of the proportion of positive reactions at low concentrations of parasite (the bold cells indicate the concentrations used to calculate the score), the definition is given in the section Material and Methods; an example of the calculation of the score is given here: the maximum hit for the score on *Plasmodium vivax* sporozoites standard is 48 reactions (12 at the dilution D, +12 at the dilution E, +12 at the dilution F and +12 at the dilution G), the score obtained with the primer pair I is 75% (36/48 = (12+12+10+2)/48).

### Absolute quantification of *Plasmodium* sporozoites

Typical amplification and standard curves derived from the dilution series performed on calibrated sporozoites standards are illustrated in the Fig 1 (panels K and L). The standard curve parameters (y-intercept, slope, r^2^ and linear dynamic) were calculated on the linear portion of each standard curve (Pearson’s coefficient r^2^>0.990). The efficiency was >80% and the value of r^2^ was >0.990 for all regression lines hence allowing an accurate quantification of the DNA target over a linear dynamic spanning from 10^4^ and 10^5^ according to the assay and standard (Tables 4 and 5).

**Table 4:** Results of the assessment of each primer set on calibrated standards of *Plasmodium vivax* sporozoites.

Primer set (%EFF and r^2^) ^a^	Parameter	Value at the indicated concentration (in Pvg equivalent per tube)							
		9,000	900	90	9	0.9	0.1	0.01	0.001
I (110%, 0.997)	Nb. Positive (%) ^b^	**9/9 (100%)**	**9/9 (100%)**	**9/9 (100%)**	**9/9 (100%)**	**9/9 (100%)**	5/9 (56%)	0/9	0/9
	Mean CP value	**17.90**	**21.18**	**24.45**	**27.70**	**30.18**	31.90	-	-
	Intra-SD ^c^	**0.10**	**0.16**	**0.17**	**0.16**	**0.42**	0.23	-	-
	Inter-SD ^d^	**0.85**	**0.71**	**0.82**	**0.80**	**0.22**	0.22	-	-
II (92%, 0.999)	Nb. Positive	**9/9(100%)**	**9/9 (100%)**	**9/9 (100%)**	**9/9 (100%)**	**9/9 (100%)**	**7/9 (78%)**	1/9 (11%)	0/9
	Mean CP value	**19.66**	**23.40**	**27.04**	**30.37**	**34.23**	**37.18**	-	-
	Intra-SD	**0.10**	**0.10**	**0.16**	**0.18**	**0.75**	**1.36**	-	-
	Inter-SD	**1.08**	**0.83**	**0.92**	**0.84**	**0.97**	**1.91**	-	-
III (110%, 0.991)	Nb. positive	**9/9 (100%)**	**9/9 (100%)**	**9/9 (100%)**	**9/9 (100%)**	**8/9 (89%)**	3/9 (33%)	0/9	0/9
	Mean CP value	**24.94**	**28.10**	**30.53**	**33.55**	**37.78**	37.56	-	-
	Intra-SD	**0.39**	**0.99**	**0.35**	**0.62**	**2.19**	0.35	-	-
	Inter-SD	**1.31**	**1.88**	**0.95**	**0.66**	**0.99**	0.56	-	-
IV (101%, 0.999)	Nb. positive	**9/9 (100%)**	**9/9 (100%)**	**9/9 (100%)**	**9/9 (100%)**	**7/9 (78%)**	2/9 (22%)	1/9 (11%)	0/9
	Mean CP value	**21.05**	**24.68**	**28.02**	**31.20**	**34.24**	35.86	35.83	-
	Intra-SD	**0.15**	**0.09**	**0.26**	**0.27**	**0.21**	-	-	-
	Inter-SD	**0.94**	**1.02**	**0.78**	**0.52**	**0.55**	1.52	-	-
V *	Nb. positive	9/9 (100%)	9/9 (100%)	9/9 (100%)	9/9 (100%)	1/9 (11%)	0/9	0/9	0/9
	Mean CP value	-	-	-	-	-	-	-	-
	Intra-SD	-	-	-	-	-	-	-	-
	Inter-SD	-	-	-	-	-	-	-	-

^a^ %EFF: efficiency (EFF) of the PCR was calculated with the formula EFF = 10^(-1/slope)^ - 1 and expressed as a percentage. An efficiency of 100% corresponds to a slope of -3.32 and means that the number of amplicons doubles after each cycle of amplification; r^2^: Pearson’s correlation coefficient expressing the intensity of the relationship between the logarithm of the concentration and the mean CP value. r^2^ varies between 0 (no correlation) and 1 (perfect correlation), a value >0.990 testify of the linearity of the method (over a defined linear range) and allow an accurate quantification. r^2^ and EFF have been calculated on the linear dynamic of each curve (bold cells).

^b^ Nb. Positive (%): number of positive reactions (amplification of the PCR DNA target) / total of reactions performed at a given dilution and corresponding percentage.

^c^ Intra-assay SD: intra-assay standard deviation (SD), calculated as the average SD of the mean CP value measured for each dilution during the same experiment.

^d^ Inter-assay SD: inter-assay standard deviation (SD), calculated as the SD of the means CP values measured during two independent experiments.

* co-amplification of primer-dimers during the PCR invalidates the calculation of the CP values and the estimation of the subsequent parameters of the best-fit line.

**Table 5:** Results of the assessment of each primer set on calibrated standards of *Plasmodium falciparum* sporozoites.

Primer set (%EFF and r^2^) ^a^	Parameter	Value at the indicated concentration (in Pfg equivalent per tube)						
		1500	150	15	1.5	0.15	0.015	0.002
I *	Nb. Positive (%) ^b^	-	-	-	-	-	-	-
	Mean CP value	-	-	-	-	-	-	-
	Intra-SD ^c^	-	-	-	-	-	-	-
	Inter-SD ^d^	-	-	-	-	-	-	-
II (84%, 0.998)	Nb. Positive	**9/9 (100%)**	**9/9 (100%)**	**9/9 (100%)**	**9/9 (100%)**	**8/9 (89%)**	2/9 (22%)	0/9 (0%)
	Mean CP value	**22.59**	**26.21**	**30.01**	**33.43**	**37.95**	38.99	-
	Intra-SD	**0.07**	**0.25**	**0.13**	**0.29**	**0.99**	-	-
	Inter-SD	**0.12**	**0.19**	**0.08**	**0.27**	**1.10**	0.40	-
III (96%, 0.996)	Nb. positive	**9/9 (100%)**	**9/9 (100%)**	**9/9 (100%)**	**9/9 (100%)**	**4/9 (44%)**	0/9 (0%)	0/9 (0%)
	Mean CP value	**25.69**	**28.67**	**32.02**	**35.98**	**37.08**	-	-
	Intra-SD	**0.24**	**0.08**	**0.42**	**1.05**	**0.61**	-	-
	Inter-SD	**0.06**	**0.17**	**0.14**	**0.38**	**1.23**	-	-
IV (97%, 0.991)	Nb. positive	**9/9 (100%)**	**9/9 (100%)**	**9/9 (100%)**	**9/9 (100%)**	**3/9 (33%)**	0/9 (0%)	0/9 (0%)
	Mean CP value	**22.60**	**25.94**	**29.35**	**33.70**	**35.73**	-	-
	Intra-SD	**0.12**	**0.06**	**0.36**	**1.10**	**1.24**	-	-
	Inter-SD	**0.09**	**0.14**	**0.11**	**0.34**	**1.22**	-	-
V **	Nb. positive	9/9 (100%)	9/9 (100%)	9/9 (100%)	0/9 (0%)	0/9 (0%)	0/9 (0%)	0/9 (0%)
	Mean CP value	-	-	-	-	-	-	-
	Intra-SD	-	-	-	-	-	-	-
	Inter-SD	-	-	-	-	-	-	-

^a^ %EFF: efficiency (EFF) of the PCR was calculated with the formula EFF = 10^(-1/slope)^ - 1 and expressed as a percentage. An efficiency of 100% corresponds to a slope of -3.32 and means that the number of amplicons doubles after each cycle of amplification; r^2^: Pearson’s correlation coefficient expressing the intensity of the relationship between the logarithm of the concentration and the mean CP value. r^2^ varies between 0 (no correlation) and 1 (perfect correlation), a value >0.990 testify of the linearity of the method (over a defined linear range) and allow an accurate quantification. r^2^ and EFF have been calculated on the linear dynamic of each curve (bold cells).

^b^ Nb. Positive (%): number of positive reactions (amplification of the PCR DNA target) / total of reactions performed at a given dilution and corresponding percentage.

^c^ Intra-assay SD: intra-assay standard deviation (SD), calculated as the average SD of the mean CP value measured for each dilution during the same experiment.

^d^ Inter-assay SD: inter-assay standard deviation (SD), calculated as the SD of the means CP values measured during two independent experiments.

* due to the lack of sample, it was not possible to perform the experiment with the primer pair I.

** co-amplification of primer-dimers during the PCR invalidates the calculation of the CP values and the estimation of the subsequent parameters of the best-fit line.

The LOD varied between 3.6 (primer sets I and II) and 360 (primer set V) sporozoites per mosquito on Pv standard, and between 6 (primer set II) and 600 (primer set V) sporozoites per mosquito on Pf standard (Table 6). The presence of *Plasmodium* was confirmed by sequencing for all standards and serial dilutions (Macrogen®, Seoul, Korea).

**Table 6:** Determination of the limit of detection (LOD) using calibrated standards of Pf and Pv sporozoites.

Primer set	LOD for the indicated species ^a^	
	Pf	Pv
I	ND ^b^	0.1 (3.6)
II	0.15 (6)	0.1 (3.6)
III	1.5 (60)	0.9 (36)
IV	1.5 (60)	0.9 (36)
V	15 (600)	9 (360)

**LOD**, limit of detection; **ND**, not determined; **Pf**, *Plasmodium falciparum*; **Pv**, *Plasmodium vivax*.

^a^ limits of detection are expressed in number of *Plasmodium falciparum* and *Plasmodium vivax* genome equivalent (Pfg and Pvg) per reaction tube, values into brackets represent the corresponding number of sporozoites per mosquito.

^b^ due to the lack of sample, it was not possible to determine the LOD of the primer pair I for Pf sporozoites. According to the score values presented in the Table 3, the LOD determined with the primer pair I should be similar to the LOD determined with the primer set II for Pf sporozoites (6 Pf sporozoites per mosquito).

The primer pair IV was further selected to quantify the sporozoite load in 49 naturally infected *Anopheles* collected along the Thai-Myanmar border (unpublished data). The mean CP value was 32.9 ± 3.0 (range, 25.1 to 35.7) and 32.3 ± 4.0 (range, 21.6 to 38.7) for Pf and Pv respectively. The range of the standard curve was appropriate for the quantification of sporozoites in most infected specimens (12 Pv infected *Anopheles* had however a mean CP value below quantification threshold which corresponds to less than 36 sporozoites per mosquito). The geometric mean of the sporozoite load was 57 (CI95% [52–60]; range, 9 to 11,428) and 137 (CI95% [132–141]; range, <36 to 273,787) sporozoites per mosquito for Pf and Pv respectively (Table 7).

**Table 7:** Descriptive statistic of the sporozoite loads in naturally infected *Anopheles* collected along the Thai-Myanmar border.

Species	Value for the indicated species				
	Nb. infected specimen ^a^	geometric mean [CI95%] ^b^	minimum ^b^	maximum ^b^	median ^b^
Pf	12	57[52–60]	9	11,428	28
Pv	37	137 [132–141]	<36	273,787	71

**CI**, confidence interval; **Pf**, *Plasmodium falciparum*; **Pv**, *Plasmodium vivax*.

^a^ Nb. infected specimen: number of *Anopheles* naturally infected with *Plasmodium*.

^b^ Geometric mean, minimum, maximum and median of the sporozoite load in *Anopheles* naturally infected with *Plasmodium*, expressed in number of sporozoites per mosquito.

**Fig 2:**
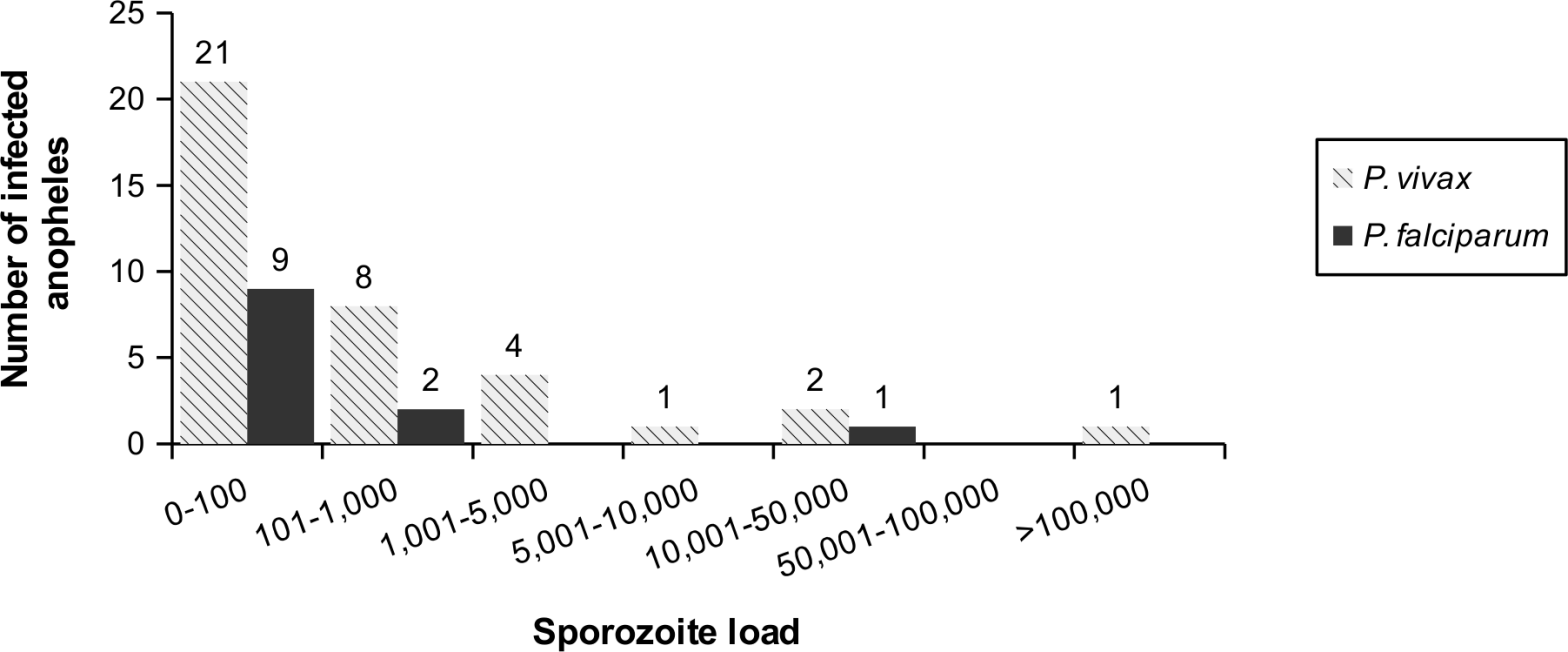
Frequency distribution of the sporozoite loads in naturally infected *Anopheles* collected along the Thai-Myanmar border. The sporozoite loads in the studied area are very low: 60% (30/49) of the infected *Anopheles* carry less than 100 sporozoites.

Overall 60% of the infected *Anopheles* carried less than 100 sporozoites and the sporozoite load seems to follow right skewed distribution (Fig 2).

## Discussion

The aim of the present work was to provide technical guidance on the best molecular method to use for the detection of *Plasmodium* in anopheline vectors with particular focus on low transmission settings. We successfully compared the performances of five primer sets with regard to their ability to *(i)* detect sporozoites, oocysts and blood stages of *P*. *falciparum* and *P*. *vivax* and *(ii)* to quantify the sporozoite load in *Anopheles* vectors.

### Discrimination between methods at low concentrations of parasite

High concentrations of parasite were accurately detected by all primers. Contrastingly, significant variations were observed at low concentrations (Table 3). The best primer set to detect blood stages was not necessarily the best primer set to detect sporozoites or oocysts. This observation confirms our hypothesis that an assay developed for the diagnosis of malaria in human can be less sensitive when transposed to the detection of *Plasmodium* in malaria vectors. The LOD of our qrtPCR assay varied from 3.6 (primer sets I and II) to 360 (primer set V) Pv sporozoites per mosquito and from 6 (primer sets I and II) to 600 (primer set V) Pf sporozoites per mosquito according to the primers used to perform the reaction.

The main limitation to discriminate between PCR assays is the Poisson’s law which applies at very low concentrations of parasite. Indeed only a certain proportion of the replicates are positive at low concentrations and the standard deviation of the mean CP value increases substantially. The pitfall of Poisson’s law can be avoided by *(i)* the use of DNA extracts combined with repetition of the PCR DNA target [40, 41], *(ii)* the multiplication of the number of reactions performed at a given dilution [39, 40] and *(iii)* by the use of a discriminative method for data analysis [39].

### The use of quantitative data to study malaria transmission

To our knowledge, this is the first report of absolute quantification of *Plasmodium* in anopheline vectors using calibrated suspensions of sporozoites. We set-up standard curves suitable for the quantification of *Plasmodium* sporozoites over 4 orders of magnitude allowing the quantification of sporozoite loads ranking from 6 to 60,000 sporozoites per mosquito for Pf and from 36 to 360,000 sporozoites per mosquito for Pv. We could generate three vials of calibrated sporozoites suspension for Pv and only one vial for Pf. However the data obtained with Pf standard were consistent with those obtained with Pv standards. The estimation of the sporozoite loads in Pf infected specimens can therefore be considered as accurate.

In the absence of calibrated sporozoite standard, some authors have set-up standard curves from suspension of plasmids containing a single copy of the PCR DNA target. However, the conversion of plasmid concentration in the standard (*i*.*e*. a known copy number of the PCR DNA target) in number of genome equivalent (*i*.*e*. number of sporozoites per mosquito) may not be possible for two reasons. In the absence of molecular data, the copy number of a given PCR DNA target in *Plasmodium* sporozoites is unknown. Moreover the efficiency of the PCR (*i*.*e*. the slope of the standard curve) is likely to be different on plasmid and sporozoite standards. If an accurate quantification of the parasite is expected, the standard curve must be set-up using the same extraction and amplification protocols for the serial dilution experiment than those used to detect *Plasmodium* in routine samples. The dilution series must be performed using appropriate *Plasmodium* standards (Pf and Pv sporozoites) and appropriate negative DNA matrix (DNA extracted from uninfected *Anopheles*). Finally the quantification of the sporozoite load (expressed as a total number of sporozoites per mosquito) must be performed without the use of a calibrator to normalize the signal.

In our study, the sporozoite loads of naturally infected *Anopheles* collected along the Thai-Myanmar border were very low (60% of the *Anopheles* carried less than <100 sporozoites). This is consistent with previous report in the area [42] and contrasts with the high sporozoite loads observed in African malaria vectors [43–45]. Interestingly our findings showed a lower sporozoite load in Pf infected mosquitoes compared to Pv infected mosquitoes (geometric means of 57 CI95% [53–61] and 137 CI95% [132–141] sporozoites respectively). Baker and colleagues reported similar sporozoite loads in Pv infected malaria vectors [42] but higher Pf sporozoite density compared to our study [42, 46, 47]. These observations suggest that both the prevalence and the density of infection become low when the transmission intensity declines. Moreover the frequency distribution of the sporozoite loads seems to follow a right skewed distribution, which contrasts with the log normal and unimodal distribution of parasite densities during asymptomatic malaria in the same area [48]. Therefore we suspect that the very low densities of infection observed in naturally infected malaria vectors might result from infective blood meals taken from submicroscopic gametocytæmia [49, 50]. Clearly much work has to be done to address the causal relationship between submicroscopic reservoir and malaria transmission along the Thai–Myanmar border.

### The role of transmission studies in epidemiological trials

An accurate detection of *Plasmodium* in vectors is crucial to evaluate the intensity of malaria transmission (*i*.*e*. the number of infective bites per person per year). Detection methods need to be sensitive and specific enough to provide an accurate measurement of vector infectivity in low transmission settings where both the prevalence and the density of infection become low [51]. Any false negative or any false positive results would results in a significant bias in the estimation of the transmission’s intensity [3, 4]. This is of major concern during epidemiological trials aiming at evaluating the effectiveness of malaria control tools (drugs or vector control) in a context of malaria control and elimination.

## Conclusion

In conclusion, the molecular detection of *Plasmodium* infection in vectors provides essential information on malaria epidemiology that is not accessible by conventional methods (either in clinical or in entomological samples). Unlike diagnosis of malaria in human, few recommendations exist for the assessment of the prevalence and density of *Plasmodium* infection in anopheline vectors. Here we generate accurate data on the performances of five primer sets in order to provide guidance for a better use of molecular methods for *Plasmodium* detection in low malaria transmission settings.

## Supporting Information

S1 Table Results of the multiple pairwise Chi-square tests between the score values obtained on Pf and Pv standards. (DOCX)
