# Supplementary material for: Comparison of the Performances of Five Primer Sets for the Detection and Quantification of Plasmodium in Anopheline Vectors by Real-Time PCR
Source: PLoS One. 2016 Jul 21;11(7):e0159160. doi: 10.1371/journal.pone.0159160 (PMC4956213; doi:10.1371/journal.pone.0159160)
Supplement: S1 Table — (DOCX) [file pone.0159160.s001.docx]

S1 Table. Results of the multiple pairwise Chi-square tests between the score values obtained on Pf and Pv standards.

| **Stage** | **Species** | **Pairwise assays** | **Bonferroni corrected p-value** | **Significance ^a^** |
| --- | --- | --- | --- | --- |
| Sporozoites | Pv | I *vs* II | 1.00E+00 | NS |
|  |  | I *vs* III | 3.48E-03 | ** |
|  |  | I *vs* IV | 1.97E-02 | * |
|  |  | I *vs* V | 9.63E-07 | *** |
|  |  | II *vs* III | 3.48E-03 | ** |
|  |  | II *vs* IV | 1.97E-02 | * |
|  |  | II *vs* V | 9.63E-07 | *** |
|  |  | III *vs* IV | 5.40E-01 | NS |
|  |  | III *vs* V | 3.28E-02 | * |
|  |  | IV *vs* V | 6.41E-03 | ** |
|  | Pf | I *vs* II | 2.34E-01 | NS |
|  |  | I *vs* III | 2.11E-03 | ** |
|  |  | I *vs* IV | 4.61E-03 | ** |
|  |  | I *vs* V | 2.69E-08 | *** |
|  |  | II *vs* III | 5.31E-02 | * |
|  |  | II *vs* IV | 9.26E-02 | * |
|  |  | II *vs* V | 3.71E-06 | *** |
|  |  | III *vs* IV | 7.95E-01 | NS |
|  |  | III *vs* V | 2.16E-03 | ** |
|  |  | IV *vs* V | 1.05E-03 | ** |
| Oocysts | Pv | I *vs* II | 8.38E-01 | NS |
|  |  | I *vs* III | 1.23E-02 | * |
|  |  | I *vs* IV | 1.23E-02 | * |
|  |  | I *vs* V | 2.39E-08 | *** |
|  |  | II *vs* III | 2.11E-02 | * |
|  |  | II *vs* IV | 2.11E-02 | * |
|  |  | II *vs* V | 5.92E-08 | *** |
|  |  | III *vs* IV | 1.00E+00 | NS |
|  |  | III *vs* V | 3.44E-04 | *** |
|  |  | IV *vs* V | 3.44E-04 | *** |
|  | Pf | I *vs* II | 1.00E-01 | NS |
|  |  | I *vs* III | 6.01E-03 | ** |
|  |  | I *vs* IV | 8.66E-02 | * |
|  |  | I *vs* V | 3.03E-08 | *** |
|  |  | II *vs* III | 1.70E-05 | *** |
|  |  | II *vs* IV | 9.13E-04 | *** |
|  |  | II *vs* V | 8.58E-12 | *** |
|  |  | III *vs* IV | 2.86E-01 | NS |
|  |  | III *vs* V | 8.16E-04 | *** |
|  |  | IV *vs* V | 2.81E-05 | *** |
| Blood stages | Pv | I *vs* II | 8.38E-01 | NS |
|  |  | I *vs* III | 6.39E-04 | *** |
|  |  | I *vs* IV | 3.16E-03 | ** |
|  |  | I *vs* V | 1.28E-07 | *** |
|  |  | II *vs* III | 3.12E-04 | *** |
|  |  | II *vs* IV | 1.66E-03 | ** |
|  |  | II *vs* V | 5.09E-08 | *** |
|  |  | III *vs* IV | 6.15E-01 | NS |
|  |  | III *vs* V | 1.93E-02 | * |
|  |  | IV *vs* V | 5.48E-03 | ** |
|  | Pf | I *vs* II | 2.30E-01 | NS |
|  |  | I *vs* III | 1.00E+00 | NS |
|  |  | I *vs* IV | 9.57E-02 | * |
|  |  | I *vs* V | 5.04E-04 | *** |
|  |  | II *vs* III | 2.30E-01 | NS |
|  |  | II *vs* IV | 6.37E-01 | NS |
|  |  | II *vs* V | 9.00E-06 | *** |
|  |  | III *vs* IV | 9.57E-02 | * |
|  |  | III *vs* V | 5.04E-04 | *** |
|  |  | IV *vs* V | 1.49E-06 | *** |

**Pf**: *Plasmodium falciparum*; **Pv**: *Plasmodium vivax*.

^a^ NS: non-significant (p-value>0.05); * : p-value<0.05 ; ** : p-value<0.01 ; *** : p-value<0.001.
